# Visual perception of axes of head rotation

**DOI:** 10.3389/fnbeh.2013.00011

**Published:** 2013-02-15

**Authors:** D. M. Arnoldussen, J. Goossens, A. V. van den Berg

**Affiliations:** Department of Cognitive Neuroscience, Section Biophysics, Radboud University Nijmegen Medical Centre, Donders Institute for Brain, Cognition, and BehaviorNijmegen, Netherlands

**Keywords:** optic flow, MST, V3A, V6, motion perception, eye movements, self-rotation, vestibular system

## Abstract

Registration of ego-motion is important to accurately navigate through space. Movements of the head and eye relative to space are registered through the vestibular system and optical flow, respectively. Here, we address three questions concerning the visual registration of self-rotation. (1) Eye-in-head movements provide a link between the motion signals received by sensors in the moving eye and sensors in the moving head. How are these signals combined into an ego-rotation percept? We combined optic flow of simulated forward and rotational motion of the eye with different levels of eye-in-head rotation for a stationary head. We dissociated simulated gaze rotation and head rotation by different levels of eye-in-head pursuit. We found that perceived rotation matches simulated head- *not* gaze-rotation. This rejects a model for perceived self-rotation that relies on the rotation of the gaze line. Rather, eye-in-head signals serve to transform the optic flow's rotation information, that specifies rotation of the scene relative to the eye, into a rotation relative to the head. This suggests that transformed visual self-rotation signals may combine with vestibular signals. (2) Do transformed visual self-rotation signals reflect the arrangement of the semi-circular canals (SCC)? Previously, we found sub-regions within MST and V6^+^ that respond to the speed of the simulated head rotation. Here, we re-analyzed those Blood oxygenated level-dependent (BOLD) signals for the presence of a spatial dissociation related to the axes of visually simulated head rotation, such as have been found in sub-cortical regions of various animals. Contrary, we found a rather uniform BOLD response to simulated rotation along the three SCC axes. (3) We investigated if subject's *sensitivity* to the direction of the head rotation axis shows SCC axes specifcity. We found that sensitivity to head rotation is rather uniformly distributed, suggesting that in human cortex, visuo-vestibular integration is not arranged into the SCC frame.

## Introduction

Eye signals and vestibular signals modulate visual responsiveness in extra-striate cortical areas (Andersen et al., 1985, 1999; Andersen, 1989; Galletti et al., 1995; Bradley et al., 1996; Shenoy et al., 1999, 2002; Takahashi et al., 2007; Angelaki et al., 2009b). Such modulations provide a necessary step toward the transformation of visual signals from retinal to other reference frames (Andersen et al., 1997), for example, to perform correct reaches when the head is tilted (Blohm and Crawford, 2007; Blohm et al., 2009). One important faculty that is served by such transformations is perception of self-motion (Britten, 2008). Self-motion specifies the changing position of the body relative to the world. Which part of the body's motion is specified depends on the sensory system that collects the motion information. The pattern of motion on the retina, or retinal flow, specifies the eye's motion through the world (Gibson, 1957; Koenderink, 1986) but our ears (Maier and Ghazanfar, 2007) and vestibular system (Angelaki and Cullen, 2008), register the motion of our head. Motion transformations are needed to align the signals from these different sensory systems for meaningful multi-modal integration (Andersen, 1997).

Thus, movement of the head and the eye relative to space are registered through the vestibular system and through visual flow on the retina, respectively. How are these signals combined into an ego-rotation percept? Eye pursuit complicates the perception of the heading direction and the direction axis of rotation relative to the head from the visual flow. For example, even at the instant when the eyes' and the head's reference frames are aligned, the eyes' horizontal pursuit velocity alters the visual flow in such a way that the center of expanding flow is horizontally displaced relative to the heading direction when the head is moved forward. Also, when the head is rotating about the roll axis, horizontal pursuit shifts the center of the circular flow on the retina in the vertical direction (Shenoy et al., 2002; Duijnhouwer et al., 2008, 2010). Thus, to find out how the head *moves* in space, retinal flow needs to be corrected for the *movement* of the eye in the head.

Regarding the neural correlate of the transformations from eye to head reference frame, it is known that in the middle temporal cortex of the monkey (area MST), extra-retinal eye-in-head rotation signals make visual self-motion signals invariant to the eye's movement-in-the-head (Inaba et al., 2007), building a *visual* representation of the translation of the head relative to the world (Bradley et al., 1996; Shenoy et al., 1999). Area MST has no known topographical structure despite local clustering of similarly tuned cells (Britten, 1998; Fetsch et al., 2010). Electrical stimulation of this area biases perceived direction of translation by the monkey (Britten and Van Wezel, 2002), showing its contribution to the heading percept. Such visual representations of head translation combine with vestibular head translation signals (Liu and Angelaki, 2009) to improve multi-modal heading discrimination (Gu et al., 2008). Visual and vestibular input concerning head *rotations* is also found in area MST of the monkey but its relation to the self-rotation percept is not clear because visual and vestibular rotation preferences are opposite in virtually all cells (Takahashi et al., 2007), perhaps pointing to an involvement in object perception (Gu et al., 2008; Yang et al., 2011).

The ventral intraparietal regions (VIP) has also been extensively linked to self-motion perception, having very similar response properties as MST, both in terms of visual flow, vestibular sensitivity (Bremmer et al., 2002; Chen et al., 2011), and its direct causal relation to the self-motion percept (Zhang and Britten, 2011).

We wondered if the neuronal organization of a cortical visual/vestibular region like MST or VIP, might be dominated by the vestibular organization. A possible visuo-vestibular integration might become apparent by a non-uniform sensitivity for visual head rotations about cardinal axes to which the semi-circular canals are optimized. If so, visuo-vestibular interaction can be optimal, as they are in a common reference frame. Such a vestibular imprint on visual self-motion sensitivity has been found in sub-cortical structures in several animals, for example the rabbit and pigeon (Simpson, 1993; Wylie et al., 1993, 1998). These structures are part of the Accesory Optic System (AOS), which is a sub-cortical visual pathway involved in image stabilization, by generation of compensatory eye movements in response to flow fields (Wylie et al., 1993).

The current study explores the possibility that a similar preference is (still) apparent in higher order cortical motion areas. If so, a specific analysis on rotations about the vestibular cardinal axes might reveal a spatially distinct responsivity of neural units. As of yet, it is unkown if cortical flow responsive regions have a similar vestibular imprint on the reference frame, given their involvement with more complex sensory-motor transformations than structures within the AOS. Yet, we hypothesize that such an organization might still be beneficial, given their involvement in the rotation of the head in space. Visual and vestibular input concerning head *rotation* is found in area MST and VIP of the monkey (Takahashi et al., 2007; Chen et al., 2011). Therefore, these areas are the main candidates to benefit from a possible vestibular organization of visual self-rotation information. Regarding MST, the study by Takahashi et al. (2007) showed visual rotation sensitivity to be scattered along the manifold, suggesting no ordening along the cardinal semi-circular axes. However, the study of Takahashi et al. (2007) mapped sensitivity to the axes of retinal rotation, not head rotation. Thus, cardinal axes of specficity might still be present for visual head rotation.

In humans, it is known that the MT^+^ complex responds to ego-rotations (Deutschlander et al., 2002; Kleinschmidt et al., 2002). Remarkably, sub-regions of human MST, V6^+^, and V3a respond to simulated *head* rotation (Goossens et al., 2006; Arnoldussen et al., 2011). Human MST shows sensitivity to vestibular information, while V6 appears not (Smith et al., 2012). Hence, in human MST more than in V6, it seems plausible that neural units responsive to the visual rotational speed of head rotation integrate visual and vestibular head rotation signals. The integration of these signals can take place in a visual or vestibular reference frame.

Human pVIP and CSv can also be considered candidates for visuo-vestibular integration, as fMRI work revealed a larger preference for self-motion in pVIP and CSv than MST (Wall and Smith, 2008), and a sensitivity to vestibular signals in pVIP (Smith et al., 2012), and to visual rotation signals in CSv (Fischer et al., 2012a).

Our simulated self-motion experiments in humans (and in many other cases) were done without real head or body movements. Thus, our studies resemble the case of a passenger in a car that has minimal proprioceptive or efference copy signals related to the driving of the vehicle and without vestibular information. Proprioceptive information is absent with the exception of those from the eye muscles. Therefore, when we speak about eye-in-head rotation signals, we do not distinguish between eye-muscle rotation and efference copy contributions. Also, in our experiments with the head stationary, vestibular signals, and proprioceptive signals regarding head rotation, i.e., the proprioceptive neck receptors, indicate absence of rotation, and we cannot distinguish between their separate contributions to the conflict with the visually simulated head rotation. Our study thus focuses on the question how visual and extra-retinal eye movement signals combine with a NULL vestibular/proprioceptive signal on self rotation. It should be noted that the vestibular system acts as a high-pass filter, contributing mainly during relatively higher frequency head motions. Hence, visuo-vestibular integration is benificial for registration of head rotation across a full range of frequencies, including sinusoidal low-frequency rotations as used in the current study.

We performed three experiments, which aimed to provide insight into these questions regarding the relation between visual and vestibular sensory signals on self-motion in humans. First, we investigated if subject's perceptual rating of self-rotation is based on the simulated head rotation or gaze rotation. Next, we performed an fMRI study to investigate the spatial organization of Blood oxygenated level-dependent (BOLD) signals to the three cardinal axes of simulated head rotation, within the head-centric region of pMST and V6^+^. We show that these sub-regions are responsive to the speed of simulated head rotation, regardless of the axis of rotation with possibly a small regional preference for particular axes of head rotation.

Finally, we performed a psychophysical study that investigated sensitivity to periodic changes in the direction of the visually simulated head rotation axis (precession). When the orientation of the precession axis with respect to the semi-circular canals was varied, we found no differences of the threshold for detection of precession, suggesting that the visual sensitivity to the directional axis of visually simulated head rotation may be uniform on the sphere.

### Experiment 1: visual axes of head rotation

#### Rationale (Experiment 1)

Neuro-physiological studies suggest, that neurons/regions sensitive to retinal rotational signals are influenced by eye rotation signals (Andersen, 1997; Inaba et al., 2007). In humans, at least one such region, pMST, uses these signals to be sensitive to the rotational speed of the head in space (Goossens et al., 2006; Arnoldussen et al., 2011). In many higher order human motion areas this region of head in space rotation is accompanied by a neighboring region sensitive to the rotation of the line of sight relative to the scene: gaze rotation (Arnoldussen et al., 2011). From these data it is unclear whether perceived rotation speed is like one or the other. Up to now, it has not been investigated if the rotational speed percept matches simulated head-in-space rotation or simulated gaze rotation.

Therefore, we started with a psychophysical study that varied the simulated rotation of a scene about the head or the eye of the subject and investigated if the rotational percept matches gaze or simulated head rotation.

#### Methods (Experiment 1)

**Figure 1:**
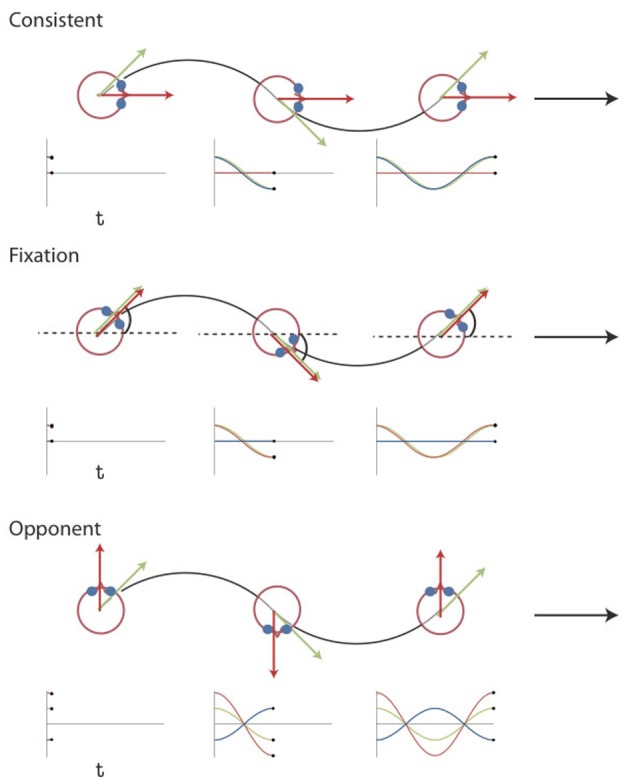
**Visual Stimuli Experiment 1 and 2.** Top view of the simulated path for the three different conditions together with plots of the gaze (green), eye-in-head (blue), and head-in-space (red) orientation at different instants in time. The flow field simulates self-motion through space in a forward direction (the global heading direction, black arrow), but along an oscillating path (black trajectory). Rotation of the eyes and head are defined relative to the global heading direction (symbol ω for the head). The gaze rotation is identical in the three conditions. At every time point, gaze is aligned with the tangent of the path (green arrow). As a result, the flow patterns on the retina remain the same. Combined with different eye-in-head positions, however, different simulated head rotations (red arrows) are defined. At the three instances depicted, the direction of motion along the path deviates maximally from the global heading direction. In the *consistent* condition, the amplitude and direction of simulated gaze rotation matches the amplitude and direction of the eye-in-head rotation. Thus, the simulated head rotation is 0. In the *fixation* condition, eye and head remain aligned with the momentary heading direction. Therefore, simulated gaze and head rotation are identical. In the *opponent* condition, simulated gaze rotation is always opposite in direction to the eye-in-head rotation. Now, the eye rotation relative to the global heading direction remains the same, but the magnitude of the simulated head rotation is doubled. Note that the angles of rotation are exaggerated for visualization purposes [adapted from Arnoldussen et al. (2011)].

We dissociated components of simulated rotation of gaze and head by combining the same retinal flow with different eye movements [Figure 1, see also Arnoldussen et al. (2011)]. The retinal flow pattern simulated a forward motion of the eye along a sinusoidal path. The simulated gaze was always aligned with the heading direction, i.e., the tangent to the path. Hence, the gaze line turned during the presentation, and caused a rotational component of flow. In the *fixation* condition the eye is stationary in the head (Figure 1). Therefore, the rotational component of the flow on the retina simulates identical rotation of gaze and head relative to the scene. In the *consistent* condition, the subject makes a real pursuit eye rotation, which matches the simulated rotation of the gaze line in direction and magnitude. Together they specify therefore no rotation of the head relative to the scene. In the *opponent* condition, a smooth pursuit is made, equal but opposite to the simulated gaze rotation. Hence, the implied rotation of the head in the scene doubles compared to the fixation condition (Figure 1).

Subjects were instructed to report the speed of rotation as a relation between themselves and the visual environment without regard to the origin of the motion being in the display or themselves. Thus, we asked subjects to judge the relative rotation between themselves and the environment, and did not quantify the amount of vection. Our instruction aimed to focus the subject's attention on the visual rotation information *per se* not the interpretation of its origin. One might therefore consider this an “imagined” form of self-motion. To what extent ratings of vection may have affected the ratings we asked for we do not know. We note though that we have no indications that significant vection occurred from debriefings of our subjects.

Observers (*n* = 5) judged whether the trial-by-trial rotation relative to the scene was higher than an internal mean based on the entire trial history. None of the subjects spontaneously reported vection, but could easily determine their amount of rotation relative to the scene. Likely, the (dynamic) edges of the relatively large display and the edges of the screen caused limited vection. The fixation condition was presented at different speed levels (*n*_*F*_ = 5) with a group-mean equal to the middle condition (*F*_ref_). One *consistent* (C) and one *opponent* (O) condition with the same simulated gaze rotation as the mean were also shown.

Stimuli were presented on a CRT screen at 25 cm distance. Observers (*n* = 5) had their heads restrained using a bite board and viewed the stimuli (angular extent: 60 × 45°) with the left eye only; the right eye was covered. Each single trial lasted 18 s, i.e., 3 rotational cycles, with a fixed frequency (*f* = 1/6 Hz) and variable amplitude (A deg: maximum angle of rotation, and corresponding peak rotational speed 2 π A f °/s). Each trial condition was randomly picked from seven stimulus conditions: five *fixation* conditions with different retinal rotational speed levels (i.e., simulated gaze rotation speeds) plus one *opponent* and one *consistent* condition, with a retinal rotational speed level equal to the middle *fixation* condition. In each trial, observers judged the speed of the perceived rotation from the flow field as either slower or faster than an internal mean, which was built up during each session. Each block of trials lasted about 10 min. In one block of trials we simulated rotation about one of the three vestibular axes: Vertical (VERT), Left-Anterior Right-Posterior (LARP), and Right-Anterior Left-Posterior (RALP) and for one of three different mean retinal rotational speed levels. Thus, each subject completed a total of nine separate experimental sessions, randomly ordered and balanced across subjects.

**Figure 2:**
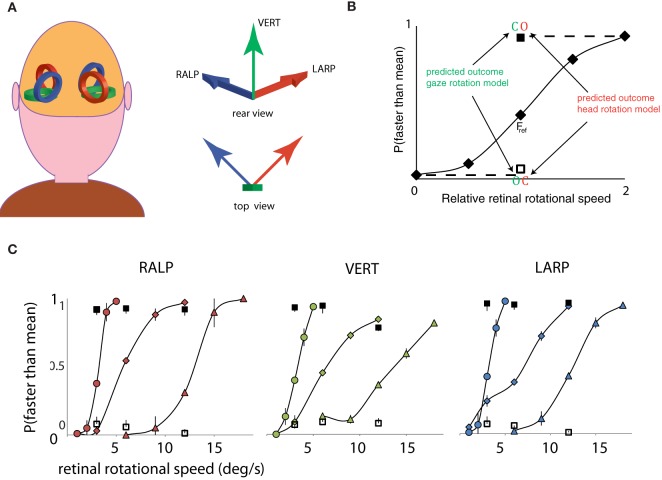
**Three axes of head rotation and results for Experiment 1. (A)** Three axes of head rotation. Rear view of the head and the illustrative location of the semi-circular canals. The semi-circular canals in left and right ear are orientated within left and right inner ear such as to be sensitive to three orthogonal axes of head rotation. The arrows show the axes of rotation for the three canal planes: the Vertical canal axis (VERT, green), the Right-Anterior Left-Posterior canal axis (RALP, blue), and the Left Anterior Right Posterior canal axis (LARP, red). **(B)** Predicted outcomes for gaze and head rotation models for Experiment 1. The results are averaged for all subjects, for three mean retinal rotational speed levels, and for three axes of rotation. The five fixation conditions (diamonds) form a psychometric curve of the probability that self-rotation is judged faster than the mean. Subjects always judged the rotational speed of the consistent condition (blank square) as slower and the opponent condition (filled square) as faster than the mean of all the stimuli. The results for the consistent and opponent condition are plotted at the corresponding retinal rotational speed level (*F*_ref_). Notice that the responses for consistent and opponent condition correspond with the responses for relative speeds at 0 and 2, respectively (indicated by dashed lines). Error bars are smaller than plotting symbols. **(C)** Results of Experiment 1, for the LARP, VERT, and RALP axis of simulated head rotation, separately. Each curve shows results of one speed range. Corresponding responses to consistent and opponent condition are given by the clear and filled square, respectively. Error bars represent standard error of the mean (SEM).

First, a psychometric curve was obtained for the simulated gaze rotation levels of the *F*_ref_ group. Secondly, we compared the response pattern of the opponent condition and the consistent condition to the psychometric curve of the fixation conditions. This procedure was repeated for three different mean speed levels and for the three axes of rotation of the semi-circular ducts. As depicted in Figure 2A, the three semi-circular ducts of the vestibular system specify three orthogonal axes of rotation. The horizontal canals specify head rotation about a vertical axis (Figure 2A, green arrow). Similarly, combinations of flow and pursuit were presented for rotation about the RALP canal axis and the LARP canal axis (Figure 2A, blue and red arrow).

#### Results/Discussion (Experiment 1)

***Rotation based on head-centric rotation, not gaze rotation.*** Figure 2B shows the proportion faster responses as a function of the peak velocity of the oscillation. Clearly, when the simulated rotation speed increased, the perceived rotation was faster. This held, irrespective of the axis of rotation and the speed-range, at least up to 20°/s. For fixating eyes, the reference frames of eyes and head are aligned throughout each trial and no distinction can be made whether the rotation was judged on the basis of the scene rotation relative to the retina or the scene rotation relative to the head. To decide between these possibilities, the manner in which eye-in-head rotation signals are combined with sensory signals is crucial. We evaluated the perceptual speed of rotation subjects reported for the consistent and opponent condition for each axis separately.

For each rotation axis and *F*_ref_ group, observers judged the consistent and opponent condition significantly different from the *F*_ref_ condition at same retinal speed level [Repeated Measures ANOVA: RALP (consistent, opponent): *F*_(1, 4)_ = 55.4; 34.4, *p* < 0.01; VERT (consistent, opponent): *F*_(1, 4)_ = 59.5; 44.4, *p* < 0.01; LARP (consistent, opponent: *F*_(1, 4)_ = 54.3; 40.5, *p* < 0.01]. More precisely, the response pattern for the consistent and opponent condition matched that of the slowest and fastest fixation condition of the *F*_ref_ group. This is clear evidence that observers' judged the rotational speed between themselves and the scene on the basis of simulated rotational motion of the head and not of the eye, in three-dimensions. We conclude that rotational components of retinal flow and eye movement signals are combined to represent the simulated rotation of the scene relative to the head, because the percept changes from no rotation (consistent) to the fastest rotation (opponent) by a mere 180° phase shift between the same rotational flow and the same pursuit movement.

First we note that natural self-rotations result in combined stimulation of visual, proprioceptive, and vestibular modalities with various efference copy signals of the movements of body parts [notably eye and head, (Andersen et al., 1997; Gu et al., 2008)]. Our presentation of rotational flow to a fixating eye with head and body maintaining a stationary orientation provides a conflicting *self-motion* interpretation; the visual system reports self-rotation from optic flow while the vestibular system and proprioceptive neck receptors report no rotation.

Depending on the extent of the conflict, averaging of- or selection between the conflicting signals may form the basis for the percept (Angelaki et al., 2009a; Fetsch et al., 2009; Lochmann and Deneve, 2011). Our results clearly reject one outcome: *selection* of the vestibular rotation signal for the percept. In that case, perceived rotation should have been zero for all conditions, because the subject's head was stationary throughout the experiment. But clearly one cannot conclude fom this that the vestibular signal can not dominate the response when real head movement is made.

Can we decide between averaging and the selection of just the visual information? A necessary step for any averaging model is the transformation between the different reference frames of the visual and vestibular sensory signals; averaging signals from different reference frames results in a signal that refers to neither. Eye-in-head movement signals serve to link the vestibular and visual reference frames.

Two possible types of averaging may occur:

1. averaging of a visual rotation signal with a *vestibular signal that has been transformed to the retinal motion reference frame* or,
2. averaging of a vestibular signal with a *visual signal, which has been transformed to represent the visual rotation relative to the head*.

We re-examined the results to distinguish between the two averaging and the visual selection possibilities. The first pursuit condition was called *consistent*. It combines eye pursuit (*E*_*h*_: eye-in-*head* rotation) with matching visual rotational flow on the retina (*E*_*s*_: eye relative *scene* rotation). The second pursuit condition was called *opponent*. It combines *E*_*h*_ with *E*_*s*_ in anti-phase (*E*_*h*_ = −*E*_*s*_). Table 1 explains the predictions of the three different models of rotation perception for these two pursuit conditions and the *fixation* condition (*E*_*h*_ = 0). For clarity we repeat that the eye received the *same* retinal flow stimulus in these three conditions.

Table 1We define the following symbols: (SCC) for vestibular rotation signals from the semi-circular canals and (SCC^*^) for a head rotation representation, from optic flow signals after subtraction of the rotation of the eye in the head.

**Table:** 

Likewise we define: (*G*) for gaze rotation signals from optic flow and, (G^*^) for the rotation relative to the eye, from *vestibular* signals to which the rotation of the eye in the head is added.			
In either case the transformation is done through an efference copy of an eye-re-head signal (*E*_*h*_).			
The first averaging model predicts that the self-rotation percept is determined by the gaze rotation: i.e., the average of *G* and G^*^ = (SCC + *E*_*h*_). This results in,			
(1) Gaze Averaging: Rotation = (*G* + (SCC + *E*_*h*_))/2.			
The second averaging model predicts self-rotation determined by the average of the vestibular rotation signal SCC and SCC^*^ = (G − E_*h*_). This results in,			
(2) Head Rotation Averaging: Rotation = (SCC + (*G* − *E*_*h*_))/2.			
Finally, the third model (selection of the rotation of the scene relative to the retina) leads to the following prediction:			
(3) Visual Selection: Rotation = *G*			
In our experiment the following constraints apply:			
(a) Because the head was stationary, SCC = 0.			
(b) The optic flow specifies the rotation of the eye relative to the scene: *G* = *E*_*s*_.			
(c) For CONSISTENT: *E*_*s*_ = *E*_*h*_			
(d) For OPPONENT: *E*_*s*_ = –*E*_*h*_			
Thus, we obtain the following table of predictions by substitution of Equation (a–d) in Equation (1–3):

**Table:** 

	**Models**		
	**Gaze averaging**	**Head averaging**	**Visual selection**
**STIMULI**			
CONSISTENT	*E*_*s*_	0	*E*_*s*_
FIXATION	0.5 *E*_*s*_	0.5 *E*_*s*_	*E*_*s*_
OPPONENT	0	*E*_*s*_	*E*_*s*_

Table 1 defines three models of multi-modal integration of rotation signals: gaze averaging, head averaging, and visual selection, and their prediction of the result of Experiment 1. (see legend Table 1). The results from Experiment 1 showed that the *opponent* condition was judged twice as fast as the *fixation* condition, and the *consistent* condition was judged as having the lowest rotation (Figure 2). Clearly, the head averaging model correctly predicts these results: perceived rotational speeds were ordered as *opponent* > *fixation* > *consistent*. This result supports the model that averages vestibular signals with visual signals on scene rotation relative to the head. Apparently, the perceived speed reflects the simulated rotation of the head, not the eye. This finding is roughly consistent with a model of head-centric motion perception (Freeman et al., 2010), as Freeman's model supposes integration of retinal speed and pursuit speed to come up with a estimate of head-velocity.

This suggests that the head-centric flow (HF) regions in the higher order motion areas may contribute particularly to the perceived speed of rotation. In Experiment 2, we investigate the discussed possibility that the visual system imposes a topography within the HF regions (Goossens et al., 2006; Arnoldussen et al., 2011; Fischer et al., 2012b) using fMRI.

We investigated whether the HF regions are equally responsive to any direction relative to the head or lignes up with the vestibular sensitivity axes.

### Experiment 2: fMRI: visual axes of head rotation

#### Rationale (Experiment 2)

Several physiological studies demonstrated cortical multi-modal integration between visual and vestibular rotation signals (Deutschlander et al., 2002; Takahashi et al., 2007) and translation signals (Angelaki et al., 2009a). In several species [goldfish (Allum et al., 1976), birds (Wylie et al., 1998), rabbit (Mathoera et al., 1999), monkey (Krauzlis and Lisberger, 1996)], visual self-motion information is known to be organized in the reference frame of the vestibular system in the sub-cortical accessory optic system (Simpson, 1993), with distinct subdivisions for visual rotation sensitivity about different axes aligned with the semi-circular canals. As of yet, a preferred reference frame if any for this mode of integration in cerebral cortex of human (or monkey) has not been demonstrated. To find out, we used fMRI to establish the representation of axes of rotation that were characteristic for the speed relative to the head. Using the set of stimuli described in Experiment 1, we previously found that when motion is shown on the retina for compensatory and anti-compensatory eye movements during simulated head rotation *without visible features attached to the head*, BOLD activity in the motion sensitive area of the human cortex (pMST) is proportional to the simulated rotation of the head. Thus, visual self-rotation in parts of area MST reflects the rotation of the head, not of the eye (Goossens et al., 2006; Arnoldussen et al., 2011). However, it is unknown if the neural units sensitive to head rotation are organized along a preferred axis of head rotation.

Hence, we re-analyzed our previously published fMRI results to investigate if the sub-region of MST that responds to the speed of simulated head rotation is spatially organized along neural units' preference toward one of the vestibular cardinal axes. Such an organization might benefit visual-vestibular interactions, that are known to take place in this area in the monkey (Takahashi et al., 2007).

#### Methods (Experiment 2)

A detailed description of the methods for Experiment 2 is given in a previous study (Arnoldussen et al., 2011). Five healthy subjects participated in the experiment. All subjects were experienced with the visual stimuli and with fMRI experiments. Subjects participated in multiple scanning sessions of about 90 min each. In all sessions, subjects were instructed to fixate the fixation point and, during pursuit, follow the fixation as accurately as possible, and pay attention to the surrounding flow. In separate experiments it was established that the pursuit (gain average = 0.9) and phase lag (average: 2.3°) were nearly perfect (Arnoldussen et al., 2011).

BOLD signals were obtained while subjects viewed wide-field presented, 3D optic flow stimuli that simulate independently varied gaze and head rotations, as described in Experiment 1. These visual stimuli allowed us to dissociate modulations of the BOLD signal toward the rotational flow speed relative to the eye, the rotational flow speed relative to the head, and the (real) eye-in-head pursuit speed.

We used wide-field projection (120 × 90° H × V) on a screen placed very near (~3 cm) the subject's head. To allow sharp vision with moderate accommodation at 3 cm the subject wore a contact lens of 30 D in the left eye. The right eye was patched. We did not quantify vection or perceived rotational speed in the fMRI experiment. On debriefing, most subjects reported no vection at all or limited vection.

Maps of V1 − V3, MT^+^, and V6^+^ were established using polar angle and eccentricity-mapping with a wedge/ring that contained expanding motion (Sereno et al., 1995; Pitzalis et al., 2006). The MT^+^ cluster was partitioned in MT and MST sub-regions, testing for BOLD responses to ipsi-lateral stimulation in putative MST (Dukelow et al., 2001; Huk et al., 2002).

All experimental runs consisted of 158 volume acquisitions. For the experimental functional scans, a block design was used. Each run consisted of 17 blocks of 9 TR's, in which all 8 conditions (consistent and fixation each at 5, 10, 20°/s; two opponent at 5, or 10°/s, Table 2) were interleaved by a rest condition (static random dot pattern). The total sequence was preceded and ended by two dummy TR's which were not analyzed. Half the runs presented conditions in reversed order to account for order effects.

**Table 2:**
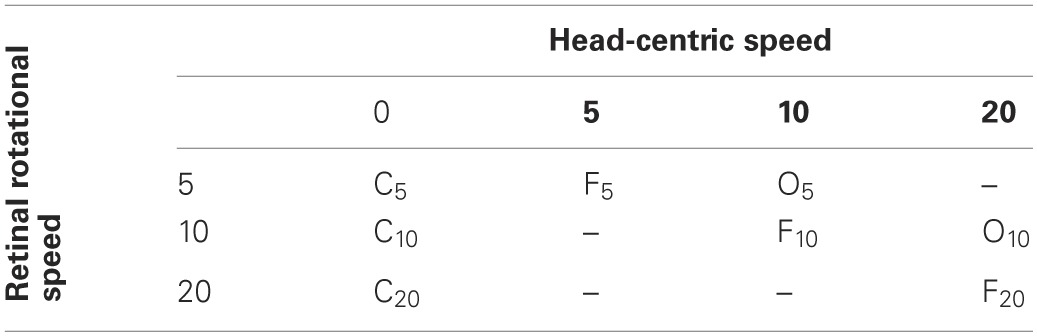
**Stimulus conditions Experiment 1 and 2**.

Brainvoyager QX (version 2.1) was used for the analysis of all anatomical and functional images (Brain Innovation, Maastricht, The Netherlands). For each subject, 17 functional scans were performed: 12 for the experimental conditions (4 per axis), 3 for the retinotopic mapping (2 for polar angle, 1 for eccentricity), and 2 for the MT/MST localizer. For every run, we discarded the first two volumes, to account for saturation effects. Subsequently, the images were corrected for 3D head motion and slice acquisition timing. The resulting time courses were corrected for low-frequency drift by a regression fit line that connects each final two data points of each static condition. Finally, a Gaussian temporal filter was applied with a FWHM of 5 data points.

To distinguish response components to head-centric and retino-centric speed of simulated rotation, we decoupled simulated gaze rotation and simulated head rotation as described in the Experiment 1. BOLD responses to *fixation*, and *consistent* and *opponent* pursuit conditions were analyzed with a General Linear Model (GLM) with predictors for pursuit speed (0, 5, or 10°/s) head-centric rotational speed (5, 10, and 20°/s) or simulated gaze rotation (0, 5, 10, and 20°/s) and one baseline activation level irrespective of stimulus condition (cf. Table 2). The slopes (Beta values) of the regression functions were tested for significance and sub-regions of MST were identified with significant linear increase of the BOLD signal with head-centric rotation speed and no significant response to the speed of simulated gaze rotation.

**Figure 3:**
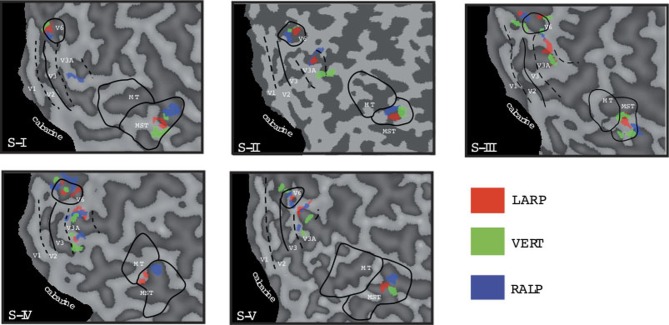
**Head-centric flow regions.** A flattened representation of the right hemisphere is shown for all five subjects. In color are shown the identified head-centric regions found using the GLM model for the LARP (red), RALP (blue), and VERT (green) axis. Global borders of the main visual areas have been demarcated.

**Figure 4:**
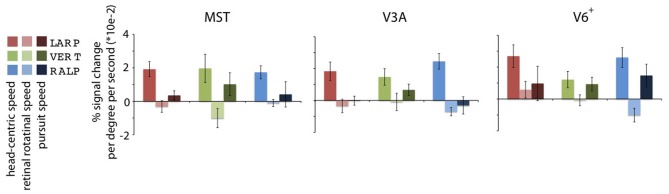
**Specificity of the headcentric flow regions.** Averaged model-based regressor estimations. For each ROI, the parameter estimations of the three identified HF sub-regions are given, averaged over all subjects. All regions are significantly modulated by simulated head rotational speed, but show no metric relation with the retinal rotational speed level. Error bars represent the SEM.

Importantly, we presented simulated head rotation for rotation about the RALP canal axis and the LARP canal axis (Figure 2A, blue and red arrow). For the previous study, head-centric regions were identified regardless of axes of head rotation. In this study, we re-analyzed the data for the three cardinal axes separately. The analyses revealed three peaks of activation for the three axes for each subject, as they are color-coded depicted in Figure 3. For each axes and each region, the weighted mean of the regression parameter, as shown in Figure 4, was calculated by:

$$\bar{x}=\frac{\sum_{i=1}^{n}\left(x_{i}/\sigma_{i}^{2}\right)}{\sum_{i=1}^{n}\left(1/\sigma_{i}^{2}\right)}$$

*n* represents the number of subjects. The variance of the weighted mean was calculated by:

$$\sigma_{x}^{2}=\frac{1}{\sum_{i=1}^{n}\left(1/\sigma_{i}^{2}\right)}$$

#### Results/discussion (Experiment 2)

***Distinct representations of vestibular axes in area MST?*** Neuronal populations within MST, V3A, and V6^+^ that are involved in the transformation of the retinal motion information into head rotation information are expected to have three properties: (1) they are significantly modulated by retinal flow; (2) they do not show a metric relation between retinal rotational speed and the BOLD response; (3) they do show a metric relation between head-centric speed and the BOLD response. Hereafter, we refer to such a region as a HF region.

We searched for HF regions that met these requirements by looking for a group of voxels that was significantly (*p* < 0.05) modulated by head-centric speed, within flow-responsive voxels in MST, V3a, and V6^+^, for LARP, VERT, and RALP axes separately.

We identified HF regions within the three ROIs in five subjects for all three axes of rotation. Figure 3 shows an overview of all identified HF regions for all subjects on a flat map representation of the dorsal part of the right hemisphere. In general, we found HF sub regions for each axis in MST, V3A, and V6^+^, in all subjects. Due to signal-to-noise ratio (SNR) limitations, we could not identify HF regions for the VERT axis in two subjects, and for the LARP axis in one subject.

Next, we averaged across subjects for each HF sub-region the parameter values for the head-centric speed, the retinal rotational speed, and the pursuit speed regressors (Figure 4). On a group level, there was no significant modulation of the BOLD signal to the retinal rotational speed [one-sided *t*-test, Bonferroni corrected (BONF) within each ROI (*n* = 3), for each axis MST_RALP_: *t*_(4)_ = −1.3, MST_VERT_: *t*_(3)_ = −1.8, MST_LARP_: *t*_(4)_ = −1.0, *p* > 0.05; V3A_RALP_: *t*_(3)_ = −1.2, V3A_VERT_: *t*_(3)_ = −1.4, V3A_LARP_: −3.2, *p* > 0.05, BONF; V6^+^_RALP_: *t*_(4)_ = 0.9, V6^+^_VERT_: *t*_(4)_ = −0.9, V6^+^_LARP_: *t*_(4)_ = −2.2, *p* > 0.05, BONF], and the pursuit speed [one-sided *t*-test MST_RALP_: *t*_(4)_ = −1.0, MST_VERT_: *t*_(4)_ = 1.7, MST_LARP_: *t*_(4)_ = 0.7, *p* > 0.05, BONF; V3A_RALP_: *t*_(3)_ = −0.8, V3A_VERT_: *t*_(3)_ = 2.1, V3A_LARP_: −0.3, *p* > 0.05, BONF; V6^+^_RALP_: *t*_(4)_ = 1.0, V6^+^_VERT_: *t*_(4)_ = 2.6, V6^+^_LARP_: *t*_(4)_ = −1.3, *p* > 0.05, BONF]. In contrast, for each HF sub-region the sensitivity for head-centric rotational speed was significantly larger than 0 [one-sided *t*-test, MST_RALP_: *t*_(4)_ = 7.0, MST_VERT_: *t*_(3)_ = 3.5, MST_LARP_: *t*_(4)_ = 11.6, *p* < 0.05; V3A_RALP_: *t*_(3)_ = 3.2, V3A_VERT_: *t*_(3)_ = 3.8, V3A_LARP_: 30.1, *p* < 0.05; V6^+^_RALP_: *t*_(4)_ = 6.4, V6^+^_VERT_: *t*_(4)_ = 3.0, V6^+^_RALP_: *t*_(4)_ = 7.7, *p* < 0.05, BONF (*n* = 3)]. Thus, the HF region identifies extra-striate cortex areas where the BOLD response is linearly related to the speed, i.e., the simulated gaze rotation, of the visually simulated rotation of the head, but not so to the retinal speed or the eye-in-head pursuit speed for that axis.

**Figure 5:**
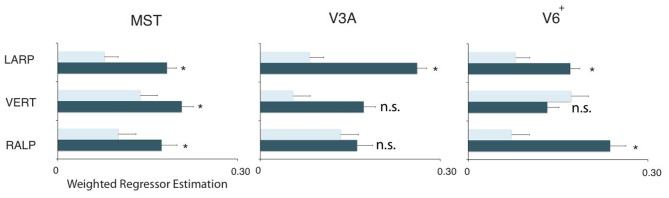
**Orthogonality of the HF regions.** Plots show the weighted average of the regressor estimations. For each sub-region and each ROI, the filled bars denote regressors for the primary axis and the open bars the average regressor of the two orthogonal axes on all three sub-regions in the three ROIs. All regions but V3A_VERT_, V3A_RALP_, and V6+_VERT_, are most responsive by a simulated head rotation around their primary axis of rotation. Error bars represent the weighted SEM. Stars represent a signicant difference (^*^p < 0.05) for a *t*-test between the beta values of the primary axis and the beta values for the orthogonal axes.

**Figure 6:**
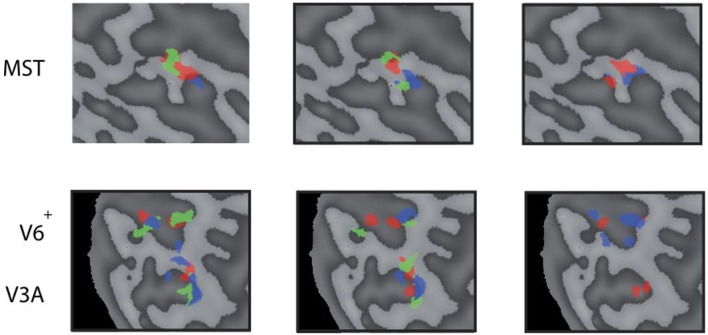
**Reproducability of the head-centric flow regions.** Subject 3 was scanned on 4 separate days three times for each axes. All results are plotted in three flat map reprentations for MST **(top row)** and V3A, V6^+^ **(bottom row)**. The results show a high variability between some sessions, and better reproduction in another; sometimes with no significant result on some of the sessions in some regions.

Next, we examine whether the spatially distinct HF regions are sensitive to orthogonal axis of rotation. At each location with peak activation for a particular axis of head-centric rotation, we determined the Beta fits to the other two axes, to check the orthogonal sensitivity of these regions to their cardinal axis of rotation, averaged over all subjects. There was significant but smaller sensitivity to the two orthogonal axes than the main (denominating) axis in all sub-regions [Figure 5; MST_RALP_: *t*_(9)_ = 5.0, MST_VERT_: *t*_(7)_ = 7.6, MST_LARP_: *t*_(9)_ = 3.9, V3A_LARP_: *t*_(7)_ = 2.7, V6^+RALP^: *t*_(9)_ = 4.4, V6^+VERT^: *t*_(7)_ = 1.1, V6^+LARP^: *t*_(9)_ = 3.8, all *p* < 0.05, BONF (*n* = 3)], except for V3A_RALP_, V3A_VERT_, and V6^+VERT^ [V3A_RALP_: *t*_(6)_ = 1.6, V3A_VERT_: *t*_(7)_ = 2.3, V6^+VERT^: *t*_(9)_ = 1.1, all *p* > 0.05, BONF (*n* = 3)]. These results hint toward a possible distinct location of neural units with a (weak) preferred responsivity to one cardinal axis. However, the variability between subjects in the location and orientation of the three blobs, raised some doubts on these findings. Therefore, we investigated the consistency and repeatability of the HF regions over sessions by repeating the experiment twice in a single subject for simulated head rotation about the three axes (Figure 6). From these results we conclude that the moderate consistency of the location of the blobs between sessions in this subject does not allow for strong conclusions regarding the spatial exclusivity of the three blobs, as shown in Figure 4. Also, the variability of the blobs within subjects (i.e., both distinct and overlapping blobs), and between subjects (no clear organization of the three blobs relative to each other or relative to space) only raise doubts concerning such a conclusion. As of yet, we consider the evidence for spatially distinct sub-regions in MST, V3A, or V6^+^ to the cardinal axes of head rotation inconclusive.

### Experiment 3: precession

#### Rationale (Experiment 3)

We did not find a strong tuning to a particular axis direction of simulated head rotation in any of the ROI's we tested in Experiment 2. This finding of nearly isotropic sensitivity for the direction of the rotation axis of the head allows for two widely different interpretations for the underlying organization at the cellular level:

1. Across the cortical surface all 3D axes of rotation are represented and cells with different tuning are completely randomly located within the ROI, which causes nearly complete coverage of all possible directions of head rotation in each sub region within the ROI. This organization has no particular reference frame.
2. There are just three populations of self-rotation sensitive units, each tuned to one of three cardinal axes. Together these detectors would form a reference frame for rotational self-motion. A nearly balanced mixing ratio within each voxel causes nearly complete coverage of all possible directions of head rotation in each sub region within the ROI. All directions of head rotation are essentially covered by the ratio's of activities between the three populations.

As we will show below, these two extreme interpretations lead to different predictions about the discrimination ability of the direction of the axis of rotation relative to the head from visual flow.

From neurophysiological studies in the macaque we know that the RF's of visual cells sensitive to self-rotation span a significant fraction (25–50%) of the visual field and are broadly tuned to the direction of the rotation axis. Some electrophysiological studies report very limited levels of clustering of cells with similar tuning (Britten, 1998; Takahashi et al., 2007) and a broad range of preferred rotation axes. Yet, so far neurophysiological data on the reference frame (if any) of cells tuned to the rotation axis of *HF* is completely lacking, because as far as we know there is no neurophysiological work that distinguishes tuning to gaze rotation from tuning to rotation relative to the head. Also we know of no psychophysical studies that directly probed the directional discrimination of head-centric rotation axes.

**Figure 7:**
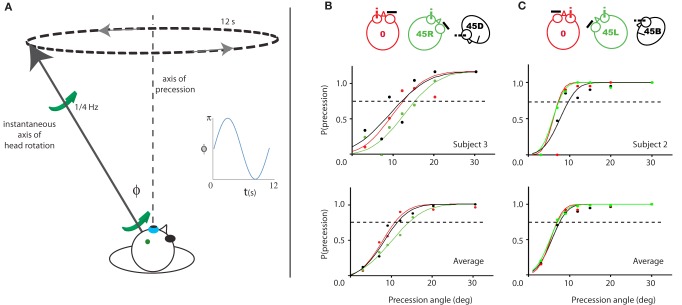
**Illustration of and results for Experiment 3. (A)** Illustration of the experiment for a given time point during the 45R, left eye condition. The instantaneous simulated head rotation axis (black line with green arrows) and the axis of precession (dashed line) are indicated. The inset shows the angle between the head rotation axis and the RALP semi-circular canal axis over time, during one precession cycle (blue line). **(B/C)** Probability of perceived precession for the left eye viewing (left column) and right eye viewing (right column) groups of subjects. One representative subject (top row) and the average of all subjects (bottom row) is shown. The color-code indicates the five different head yaw-pitch angles (0, 45L/R, 45D/U). For neither viewing eye, there is a significant difference in their precession detection threshold between the three head positions. Dashed line marks 75% precession detection threshold.

Thus, we decided to conduct an experiment in which the subject had to discriminate between a fixed axis rotation relative to the head and a variable axis of rotation; more specifically, a condition where the axis of rotation relative to the head precessed about the fixed axis at some angle (α) of which the threshold was determined (Figure 7A).

We expect that depending on the above two types of cellular organization the detection threshold for the precession will be constant on the sphere of all possible head rotation directions (model 1), or anisotropic (model 2).

First we present a simple model to estimate anisotropies of the threshold for perception of precession, which might arise from two plausible reference frames [aligned with semi-circular canals (SCC) axes or, roll, yaw, pitch] of cardinal directions for head-centric rotation perception (SCC or oculo-centric).

We assume that rotation sensitive detectors have a large receptive field covering about 50% of the visual field and have a shallow tuning to the direction of the axis of *rotational flow relative to the head.* For short we will denote this in the sequel as “preferred direction of head rotation.” Because the tuning is to a direction of the axis, the tuning function is defined on an angular (i.e., periodic) measure and the appropriate tuning function is VonMises. The angle “Φ” of this function is defined as the angular difference between the preferred axis direction and the (instantaneous) stimulus axis direction (Figure 7A illustrates this for the RALP axis of the SCC).

When the visual detectors for head rotation are arranged along the three cardinal axes of the vestibular reference frame, we note that the precession of the simulated head rotation is detected through a *modulation* of the activity in the three detectors. For a fixed axis direction the activity is constant in time. The activity in each detector is proportional to the component of the simulated head rotation along the detector's preferred axis of rotation:

$$\text{VonMises}(\Phi )=\;\frac{e^{\cos(\frac{\Phi }{s})}}{2\pi *I_{0}(\frac{1}{s})}$$

where Φ denotes the angle in radians between simulated head rotation axis and the cardinal axis of the detector, “*s*” the tuning width parameter of the detector in radians (*s* = 1 in our simulations), and I_0_ the modified Bessel function of the first kind. For fixed axis rotation it is constant, and the computed activity of a detector is always constant (VonMises(Φ)).

In contrast, the precession movement modulates the activity in time, because Φ varies according to the location of the precession axis relative to the preferred axis (e.g., perpendicular to one of the SCC planes) and the angle of the simulated head rotation axis that deviates from the precession axis by the fixed angle of precession. Hence, each detector contributes to the detection of precession by an amount equal to the integral of its *absolute activity difference relative to the mean* across one cycle of the precession movement. For each axis of precession, the computation of this modulation amplitude was done for all three cardinal axes and the root-mean-square of these three modulation amplitudes was the resultant *discrimination activity* for one particular location of the precession axis relative to the three cardinal axes. In our experiment five different head orientations were used. We computed the possible outcomes for this experiment, based on the assumption of three cardinal axes aligned with the axes of the semi-circular canals, for a spherical grid of Fick angles of simulated head rotation with a spacing of 5°.

**Figure 8:**
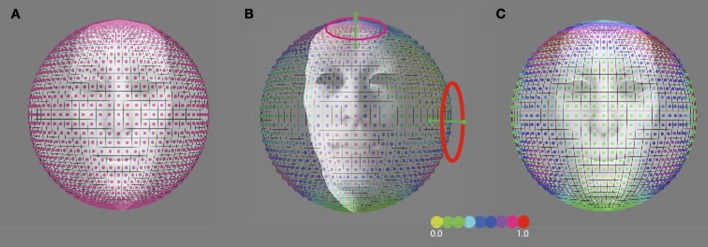
**Predicted precession threshold angles for three different models of precession coding. (A)** The uniform distribution model. Each “pixel” on the spherical surface indicates one direction axis of head rotation. The colored dot in each pixel denotes the normalized activity evoked in the model by this direction of head rotation. High activity means a low precession threshold and vice versa. Acitivity is based on the modulated depth of the entire population. The preferred axis direction of the detection forms a uniform distribution across the sphere. **(B)** The vestibular cardinal-axis model. Activity is based on the root-mean-square of the modulation depths in the three VonMises tuned detectors to the semi-circular canal axes (VERT, RALP, LARP). **(C)** The roll-pitch-yaw model. Activiteit is based on the root-mean-square of the modulation depth in VonMises tuned detectors to the roll, pitch, and yaw axes.

The discrimination activity is shown on a 3D sphere in Figure 8B, for the vestibular cardinal axes model (RALP, LARP, VERT axes), and in Figure 8C for the oculo-centric-model (ROLL, PITCH, YAW axes). The color codes the discrimination activity for the given orientation of the axis of precession on the sphere about the head and a fixed precession angle of 1°. The activity is normalized relative to the maximum of all simulated directions.

For independent noise in the detection channels one expects a threshold discrimination inversely proportional to the magnitude of the (color-coded) discrimination activity. Thus, for any frame using just three cell types one would expect, that discrimination of the axis direction of rotation is least for the cardinal axes and best in between two cardinal axes (Wylie et al., 1998).

In contrast, if the channels for the perception of head rotation are uniformly distributed on the sphere one may expect a discrimination threshold that does not depend on axis direction (Figure 8A). To find out we investigated the discrimination performance for the axis of self-rotation, for three different axes of rotation relative to the head but the same axis of rotation relative to the eye.

#### Methods (Experiment 3)

We presented two groups of subjects the same wide-field stimuli on the screen to the left eye (*n* = 4) or to the right eye (*n* = 4) for three head orientations:

1. Straight ahead (0),
2. turned 45° right (left eye viewing, 45R), or 45° leftwards (right eye viewing, 45L),
3. tilted 45° forward (left eye viewing, 45F) or backwards (right eye viewing, 45B).

The non-exposed eye was patched. Subjects maintained gaze on the screen's central fixation ring for each head orientation. Thus, the retinal stimulation by the precession stimulus was identical for all head orientations.

We presented on the screen visual flow (~60 × 45°, *h* × *v*:) that combined simulated motion along the viewing direction at 1.5 m/s with simulated rotation about an axis through the eye, which was chosen at a variable angle relative to the fixation point (i.e., close to the roll axis of the eye). The rotation axis was either stable throughout the trial or it precessed about the fixation direction at 1/12 Hz (precession angles: 0, 3, 7, 9, 12, 15, 20, and 30°/s). Sinusoidal simulated head rotation at 1/4 Hz with a peak velocity of 6°/s was shown. To prevent the detection of non-zero precession on the basis of flow elements that cross the fixation direction, zero precession-angle stimuli were always presented for an axis at a 15° deviation of the roll axis, randomly chosen into the 3, 6, 9, or 12 ‘o clock directions. During the 12 s trial, three cycles of rotation and one cycle of precession were shown.

Subjects were asked to discriminate between oscillatory flow about a single, stationary axis and oscillation about an axis that precessed at a fixed angle with respect to the axis through the fixation point.

#### Results/discussion (Experiment 3)

As shown in Figure 7, the probability of detection of the precession increases as a function of the precession angle. The psychometric curve is based on the scores of a single (upper panels) and all four subjects of each group (lower panels). The left panels show the results for the group with the left eye viewing (Figure 7B), the right panels of the other group with the right eye viewing (Figure 7C). For all three head orientations, the 75% threshold is about 12° for the left eye subject pool, and 7° for the right eye subject pool. There were no significant differences between the precession thresholds for the different orientations of the head [RM ANOVA, left/right eye pooled, main effect head orientations (0°, 45° upwards/downwards, 45° leftwards/rightwards): *F*_(2, 14)_ = 1.8, *p* = 0.20].

We compared our results with outcome for three models of head rotation organization, as depicted in Figure 8: a model that is based on a uniform distribution of axes sensitivity of neural units over the sphere (Figure 8A), a model that is based on an organization of neural units along the three vestibular cardinal axes (Figure 8B), and a model that is based on an organization of neural units along the oculo-motor rotations, the yaw, roll, pitch-model (Figure 8C). In the vestibular or oculo-centric axes models we would expect a ratio of discrimination thresholds between 45° up (or down) and the 45° left (or right) axes of more than 3, while the ratio between the observed thresholds never exceeded a factor 1.3 (left eye viewing group). Thus, the cardinal axes organization appears an unlikely model to explain human performance on this task.

Our results are more in line with an organization without cardinal axes where the axis of head rotation is encoded by the population response of multiple detectors that are tuned to different rotation axes distributed uniformly on the sphere of all possible head rotation axes, as in Figure 8A. In such an arrangement one would expect no differences between the precession thresholds as a function of the head's orientation.

Possibly the detection of the precession angle is limited by field of view? The eye brows for the head 45° down condition compared to the straight-ahead condition (left eye viewing group) covered the upper 15° of the view on the screen. No such limitation occurs when the eye in the head is turned temporally. Rather, the field increased compared to the straight-ahead orientation of the head because the nose covered less of the screen on the medial side. Field-size effects would predict therefore an increase of the threshold for the head 45° down and a threshold decrease for the head 45° right orientations, in contrast to our observations. Also, in the right eye viewing group we did not find evidence for a difference consistent with an effect of field of view limitations. We conclude that there may be true differences in precession threshold in some subjects depending on the axis direction (e.g., subject 3 in the left eye viewing group) but these are idiosyncratic and small relative to the expected differences for a true cardinal axis model. Thus, we cannot find evidence for a dependency of the precession threshold on the location of the precession axis relative to the cardinal axes of the semi-circular canals.

## General discussion

Human observers can judge their heading despite confounding eye movements. They do so by integration of retinal motion and extra-retinal eye movement signals (Lappe et al., 1999; Britten, 2008). Movements of the head and the eye relative to space are registered through the vestibular system and through optical flow, respectively. In this study, we set out a series of experiments that investigate how visual rotational flow is perceived and how it is processed and may be combined with the vestibular SCC signal to establish a coherent percept of head rotation.

### Experiment 1: models of visuo-vestibular integration

Vision is the primary sensory modality for spatial orientation in human and non-human primates. Its superior spatial resolution in the foveal part appears to “capture” contributions from other modalities (like audition) into the visual reference frame for the control of e.g., eye movements (Woods and Recanzone, 2004). On the other hand the superior temporal resolution of the auditory system sometimes “captures” the contribution by the visual modality e.g., for counting events (Shams et al., 2000; Fendrich and Corballis, 2001). These observations line-up with optimal integration models following Bayesian statistics (Battaglia et al., 2003; Deneve and Pouget, 2004). When visual spatial resolution drops, the Bayesian promotion of the most reliable signal may no longer favor vision (Wylie et al., 1998). Signal resolution drops e.g., when the visual signal is transformed to another reference frame like the head-centric frame.

In Experiment 1, we show that humans judge their rotation relative to a scene on the basis of head-centric motion signals. The responses reveal that the rotational retinal flow is transformed by eye-pursuit signals to a head-centric motion signal that could be averaged with vestibular rotation signals (Figures 2B,C). The data are not consistent with a model that averages gaze rotation from retinal rotational flow with vestibular signals that are transformed through eye pursuit signals to arrive at an estimate of the rotation of the gaze line. We conclude that the flow on the retina is combined with efference copy signals of eye-in-head rotation to recover the *head's translation* ánd *rotation*, for the percept of self-motion. Our conclusion is consistent also with a model of eye-pursuit and visual motion integration (Freeman et al., 2010) that explains many previous psychophysical studies on this subject. Our work extends this conclusion to the domain of self-motion perception for 3D axes of rotation in wide fields of view.

Apparently, superior precision of the visual system not necessarily renders dominance to its reference frame over the vestibular (or head) reference frame in this case and suggests that the head reference frame is the preferred frame for speed of self rotation perception.

### Experiment 2: responsiveness to cardinal axis of rotation in head-centric regions

Visual self-motion information is known to be organized in the vestibular reference frame in the accessory optic system (Simpson, 1993), with distinct subdivisions for visual rotation sensitivity about the axes aligned with the semi-circular canals. In a previous fMRI experiment, we found a sub-region of MST, V3A, and V6^+^ to be responsive to the rotational speed of the head in space (Arnoldussen et al., 2011). This means that these cortical regions are sensitive to rotation relative to the head, similar as the semi-circular canals. Neurons within MST are responsive to both visual and vestibular signals (Duffy, 1998; Bremmer et al., 1999). Therefore, we wondered whether the visual head-centric speed responsiveness might be organized in a vestibular format, meaning that simulated head rotation about different vestibular cardinal axes activates spatially distinct neuronal populations. We did not find evidence for this. Our data indicated overlapping distributions for sensitivity to the three cardinal axes of the vestibular system (Figure 4).

This finding seems in line with a neurophysiological study on visual-vestibular tuning of rotation for neurons in MSTd (Takahashi et al., 2007), showing a rather uniform distribution of visual and vestibular tuning to rotational axes. Because this study did not distinguish between axes of gaze rotation and axis of head rotation, as the monkeys were fixating during all trials, no clear conclusions can be drawn about the cellular organization of head-centric tuning in cortex as of yet.

### Experiment 3: uniform distribution of head rotation sensitivity

The largely overlapping activation by orthogonal rotation axes means that many voxels are equally sensitive to all directions of the simulated head rotation axis. This observation in itself does not exclude a possible rate coding by neural units with sensitivity to rotation about just three cardinal axes aligned with the vestibular system or an oculo-centric system. Such an organization would result in a non-uniform distribution of sensitivity to precession of the head's rotation axis, as illustrated in Figure 8B. In Experiment 3, we investigated whether the distribuion of head rotation sensitivity is uniformly distributed along the sphere (Figure 8A), or shows a preference reminiscent of either a vestibular cardinal axes based (Figure 8B), or roll, yaw, pitch-system based (Figure 8C) organization. The threshold precession-angle varied too little with the direction of the precession axis to lend credibility to any of the cardinal axes models. Thus, we find no evidence for an organization in cortex as in the AOS.

### Rate coding vs. population coding

Within the AOS, the preferred response of cells is clustered around the SCC cardinal axes (Simpson, 1993). In this system, as in the vestibular semi-circular canals (Leonard et al., 1988), the axes of head rotation is represented in a rate coding, in which the response rate of units clustered around the three orthogonal axes of rotation define the magnitude and direction of the self-rotation in 3D space (Wylie et al., 1998). The common reference frame allows for optimal and fast visuo-vestibular convergence of rotational signals. Here we investigated if such a rate coding is preserved in higher order cortical areas that are responsive to visual and vestibular rotation signals. We found no evidence for a rate coding of head rotation in human subjects. Rather, rotation perception sensitivty was uniformly distributed along the sphere, pointing to a population coding (Figure 8A). Transformation of the rate-coded vestibular signals toward a population coding might be more time-consuming and noisy, but can provide benefits for the complex sensory-motor computations that take place in cortex, e.g., selection of the heading direction at the expense of rotational information (Lappe et al., 1999), anticipation of object-motion trajectories despite self-movements (Land and McLeod, 2000), and spatial transformations of visual self-motion information from an eye to a body reference frame during head turns (Crowell et al., 1998).

### Vection

In all three experiments, debriefing reports of vection indicated very limited levels of vection in nearly all subjects, but was not quantified psychophysically. Regarding our fMRI results, it has been shown that vection can specifically evoke distinct BOLD responses, but mainly in higher order regions including the middle temporal gyrus, the right central sulcus, and the precuneus not evaluated in Experiment 2 (Wada et al., 2011). We note that vection is not a prerequisite for the perception or neuronal computation of the changing gaze line or straight ahead relative to the scene. It seems to us therefore likely that our results generalize to the more general condition of real head movements. Yet we agree that it is a question open to further investigation to what extent our expectation will hold for real-motion and the stimulation of the vestibular system.

## Conclusions

The visual percept of self-rotation is based on the flow relative to the head suggesting that the head's rotation is perceived rather than gaze rotation. This is true for conditions in which visual and vestibular signals give conflicting information about the rotation of the head (i.e., visual rotational flow with a stationary head), implying a transformation to the vestibular (head-centric) reference frame. An analysis of the cortical activation in the head-centric region in MST, V3A, and V6^+^ reveals that responsiveness to head rotation is not spatially distinct for rotation about different cardinal axes. Finally, we found no evidence for a preferred sensitivity to visual head rotational signals along axes aligned with the semi-circular canals.

### Conflict of interest statement

The authors declare that the research was conducted in the absence of any commercial or financial relationships that could be construed as a potential conflict of interest.
